# Supplementary material for: SyNDI: synchronous network data integration framework
Source: BMC Bioinformatics. 2018 Nov 6;19:403. doi: 10.1186/s12859-018-2426-5 (PMC6219086; doi:10.1186/s12859-018-2426-5)
Supplement: Supplementary file 5 — Files of the example presented in Additional file 4. This additional file is a zip package that contains all files used in Additional file 4. Detailed descriptions of each of these files are presented in a README file included in this zip package. (GZ 30411 kb) [file 12859_2018_2426_MOESM5_ESM.gz › SyNDITools/Meme2Fimo/meme_4.9.0_4.tar.gz!/meme_4.9.0/doc/fasta-format.html]

FASTA Format


# Sequence file format

Various MEME Suite programs require as input a file containing
protein or DNA sequences. These input files must be in FASTA
format. The format is very simple. Every entry consists of a
sequence identifier (ID), an optional comment (COMMENT), and a
sequence (SEQUENCE). The format looks like this:

```
        >ID COMMENT
        SEQUENCE
```

The special character ">" marks the beginning of a new sequence.
The ">" character is followed immediately by the sequence
identifier. The rest of that line is occupied by the optional
comment. Subsequent lines contain the sequence itself.

Some rules about representing sequences:

- Case doesn't matter. MEME Suite converts everything to
  uppercase.
- White space (spaces and newlines) within the sequence are
  ignored.
- Characters should be from a valid
  alphabet

Here is an example of three sequences in FASTA format:

```
      >ICYA_MANSE 
      GDIFYPGYCPDVKPVNDFDLSAFAGAWHEIAKLPLENENQGKCTIAEYKY
      DGKKASVYNSFVSNGVKEYMEGDLEIAPDAKYTKQGKYVMTFKFGQRVVN
      LVPWVLATDYKNYAINYNCDYHPDKKAHSIHAWILSKSKVLEGNTKEVVD
      NVLKTFSHLIDASKFISNDFSEAACQYSTTYSLTGPDRH

      >LACB_BOVIN 
      MKCLLLALALTCGAQALIVTQTMKGLDIQKVAGTWYSLAMAASDISLLDA
      QSAPLRVYVEELKPTPEGDLEILLQKWENGECAQKKIIAEKTKIPAVFKI
      DALNENKVLVLDTDYKKYLLFCMENSAEPEQSLACQCLVRTPEVDDEALE
      KFDKALKALPMHIRLSFNPTQLEEQCHI

      >BBP_PIEBR 
      NVYHDGACPEVKPVDNFDWSNYHGKWWEVAKYPNSVEKYGKCGWAEYTPE
      GKSVKVSNYHVIHGKEYFIEGTAYPVGDSKIGKIYHKLTYGGVTKENVFN
      VLSTDNKNYIIGYYCKYDEDKKGHQDFVWVLSRSKVLTGEAKTAVENYLI
      GSPVVDSQKLVYSDFSEAACKVN
```
